# Unicornuate Uterus With a Non-communicating Rudimentary Uterine Horn Treated via Laparoscopic Resection With Preservation of Both Ovaries and Fallopian Tubes: A Case Report and Review of the Literature

**DOI:** 10.7759/cureus.79562

**Published:** 2025-02-24

**Authors:** Asma Alqarni, Nourah Saeed Alqahtani, Mohamed Al-Shehri, Hala A Abdelhady

**Affiliations:** 1 Obstetrics and Gynecology, King Saud Medical City, Riyadh, SAU; 2 Obstetrics and Gynecology, Prince Sultan Military Medical Hospital, Riyadh, SAU

**Keywords:** dysmenorrhea, magnetic resonance imaging, müllerian anomaly, rudimentary horn, unicornuate uterus

## Abstract

A unicornuate uterus with a non-communicating rudimentary horn is a variant of the unicornuate uterus that is associated with gynecologic and obstetric complications. Dysmenorrhea, infertility, recurrent abortion, and acute or chronic pelvic pain are the most common presenting complaints. We report a case of a 21-year-old single girl who presented with dysmenorrhea since she began menarche. Magnetic resonance imaging and three-dimensional ultrasonography were performed, revealing a unicornuate uterus with a left-sided, non-communicating rudimentary horn. The patient underwent laparoscopic resection of the left non-communicating rudimentary horn. The presence of a left-sided, non-communicating rudimentary horn measuring 4.0 x 2.0 x 2.0 cm with secretory endometrium, which tested negative for malignancy, was confirmed via histopathologic investigation. The patient became asymptomatic after follow-up at the gynecologic clinic. The early diagnosis of a unicornuate uterus with a non-communicating rudimentary horn is crucial to prevent any complications. A thorough preoperative assessment is an extremely vital step to decrease the rate of intraoperative complications. High laparoscopic skill levels are important for the effective treatment of a unicornuate uterus with a non-communicating rudimentary horn.

## Introduction

Unicornuate uterus is a Müllerian anomaly characterized by failed or abnormal development of one of the two Müllerian ducts, which results in the presence of a normal Müllerian duct on one side and a rudimentary or absent duct on the contralateral side, which fails to reach or elongate the urogenital sinus responsible for forming the lower third of the vagina [1-3]. Unicornuate uterus occurs in approximately one in 1,000 to one in 5,400 women, and in 74-90% of them, a rudimentary uterine horn can be present [4,5]. This rudimentary uterine horn can be non-cavitary in 33% of the patients, cavitary communicating in 10% of the patients, and non-communicating, with a functional endometrium, in approximately up to 55% of the patients [5,6]. 

The differential diagnoses of a unicornuate uterus with a non-communicating rudimentary horn include a septate uterus with an obstructed horn, a bicornuate uterus with an adenomyosis or large cavity, and a cavitated accessory mass with a normal uterus or degenerative myomas [7]. The mean age of clinical presentation in case of a unicornuate uterus with a non-communicating rudimentary horn is in the early twenties, and patients typically present with dysmenorrhea and either acute or chronic dyspareunia or pelvic pain that fails to respond to medical management; however, they can also present with ectopic pregnancy (25%), pelvic mass (20%), pelvic pain (20%), and primary infertility (15%) [6,8].

Regarding the diagnosis, magnetic resonance imaging (MRI), which has a sensitivity of 75-100% and is preferred for diagnosing a unicornuate uterus with a non-communicating rudimentary horn, is mainly useful in detecting the degree of myometrial connection between the unicornuate and non-communicating horn and the presence of a functional endometrium within the rudimentary horn [9]. However, a study found that three-dimensional (3D) ultrasonography was found to be more sensitive and specific than MRI, with a sensitivity of ≥98% and specificity of 100% [10]. However, the use of hysterosalpingograms is limited in virgin or extremely young women, and they cannot accurately differentiate the subtypes of uterine anomalies [9]. Surgical resection can relieve pelvic pain and dysmenorrhea, optimize fertility, and prevent endometriosis, miscarriage, and ectopic pregnancy. Hence, due to these reasons, it is considered the standard treatment for a non-communicating rudimentary horn [11].

## Case presentation

A 21-year-old single woman who had no reported medical or surgical history presented with dysmenorrhea that had not responded well to medical therapy since she began menstruating. The patient had made emergency hospital visits for injectable paracetamol or nonsteroidal anti-inflammatory drugs, which provided symptomatic relief for severe dysmenorrhea (menstrual cramps and pain during menstruation lasting around 5-10 days), along with associated symptoms such as occasional nausea and vomiting. Her menstrual cycle started at the age of 13, which was normal. The duration of the menstrual cycle was seven days, and the volume of menstrual blood loss was normal. The patient’s menstrual cycle occurred every 28-30 days. She was scheduled for further clinical assessment at the gynecologic clinic.

**Figure 1:**
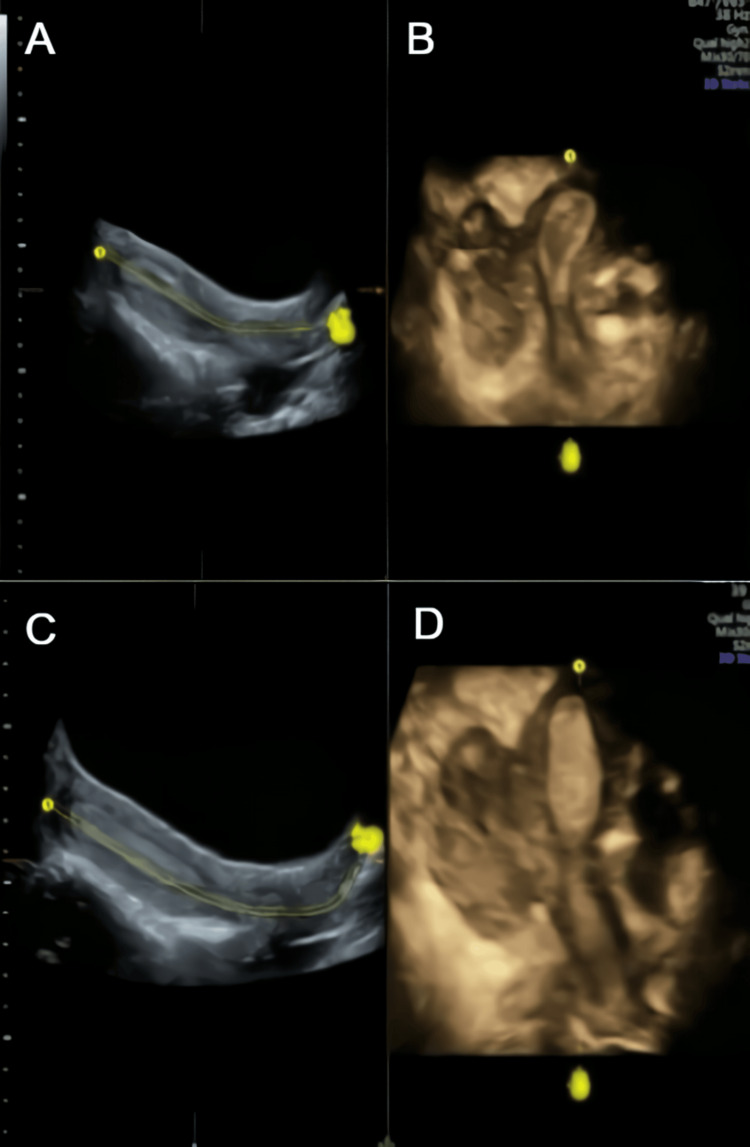
Three-dimensional ultrasonography findings. (A-C) Sagittal view showing a unicornuate uterus with a non-communicating rudimentary horn that was not separated from the uterine surface. (D) Sagittal view showing two separate endometrial cavities that indicated a unicornuate uterus with a non-communicating rudimentary horn.

**Figure 2:**
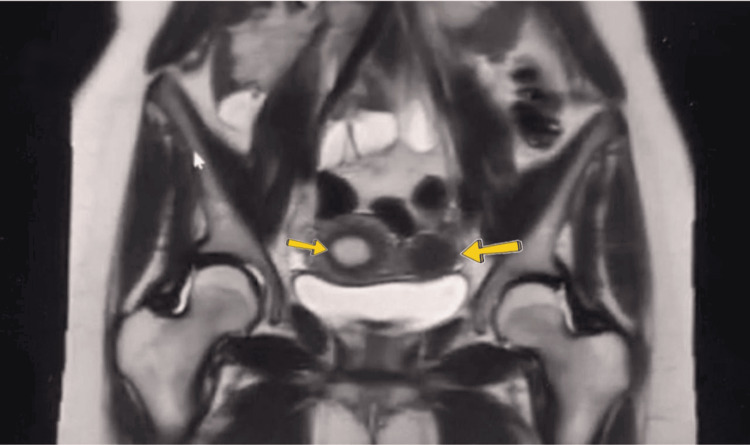
Pelvic MRI revealing both tubes and ovaries connected to the unicornuate uterus (right arrow) and the presence of a non-communicating rudimentary horn (left arrow). MRI: Magnetic resonance imaging

A 3D transabdominal ultrasonography performed by a senior sonographer showed a normal cervix with a unicornuate uterus and a left-sided, non-communicating rudimentary horn that was not separated from the uterine surface. The endometrial cavity of the unicornuate uterus was not communicating with the endometrial cavity of the rudimentary horn but the outer surface of the unicornuate uterus, and the rudimentary horn was not separated, which was later confirmed laterally by a laparoscopic procedure (Figures 1A-1D). Two ovaries were observed; the left ovary had polycystic ovarian morphology, whereas the right one looked normal. Transvaginal ultrasonography and hysterosalpingogram could not be conducted because the patient was single and not married. Pelvic MRI revealed that both tubes and ovaries were connected to the unicornuate uterus, and there was a non-communicating rudimentary horn (Figure 2). There were no signs of renal agenesis, and both kidneys looked normal.

Based on the clinical presentation and the findings on 3D ultrasonography and MRI, the patient was diagnosed with a unicornuate uterus with a non-communicating rudimentary horn. The patient underwent laparoscopic extirpation of the left non-communicating rudimentary horn. By using the LigaSure vessel sealing device (Johnson & Johnson MedTech, US), the pelvic wall adhesion on the left side of the rudimentary horn was carefully dissected step-by-step until it was completely separated, taking special care to preserve the ovarian artery. The left non-communicating rudimentary horn was also dissected using the LigaSure device, following a step-by-step approach until complete separation was achieved. A small incision of approximately 2 cm was made between the unicornuate uterus and the rudimentary horn. No bleeding occurred during the procedure.

The laparoscopic procedure confirmed the presence of a left-sided, non-communicating rudimentary horn that was not separated from the unicornuate uterus. The right tube and ovary were visualized and looked normal. Meanwhile, the left tube was not identified, and the left ovary had a polycystic ovarian morphology. Two days after the procedure, the patient’s clinical condition and vital signs stabilized, and she was discharged in good condition. The patient was asymptomatic after the procedure, and her quality of life improved when she visited the gynecologic clinic during follow-up.

**Figure 3:**
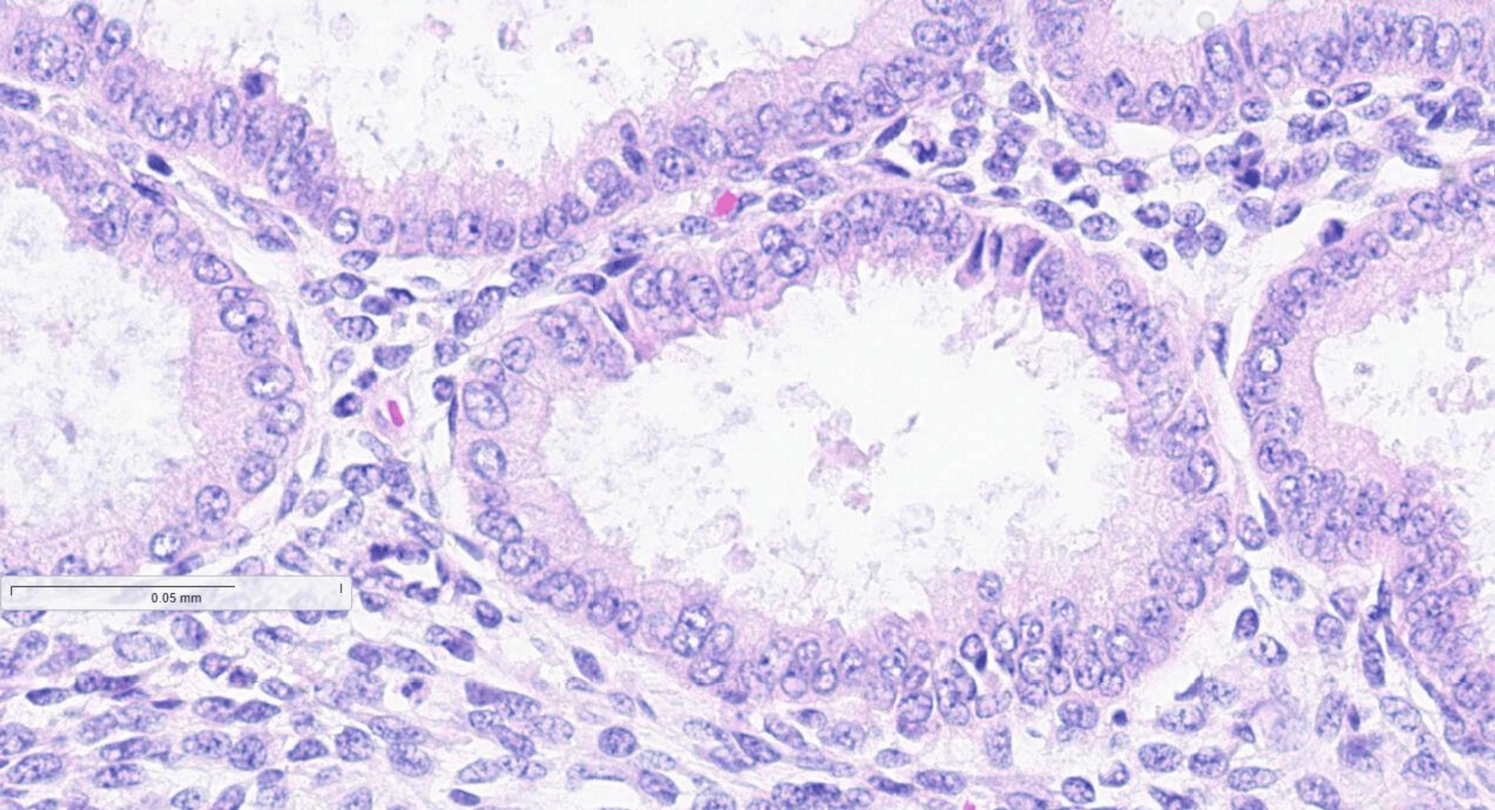
Histopathological sample showing secretory endometrium.

Histopathological investigation revealed that the left-sided, non-communicating rudimentary horn measured 4.0 × 2.0 × 2.0 cm and presented with a secretory endometrium that tested negative for malignancy (Figure 3). During the follow-up examination at our clinic after four weeks of surgery, the patient was asymptomatic and did not further complain of symptoms such as dysmenorrhea and chronic pelvic pain.

## Discussion

Congenital uterine anomalies are the most prevalent congenital abnormalities affecting the female reproductive system [12]. Septal resorption, fusion, and organogenesis are essential processes in normal development and Müllerian duct transformation. Around the 20th week of gestation, midline tissue resorption initiates the formation of the uterus, fallopian tubes, and cervix [13]. In genetic male embryos (46, XY), this process is inhibited by the production of anti-Müllerian hormone and testosterone. Conversely, in genetic female embryos (46, XX), the absence of the Y chromosome allows the Müllerian ducts to develop into these reproductive structures [14]. Early organogenesis dysfunctions resulting in one or both Müllerian ducts being absent can lead to the development of uterine hypoplasia/agenesis or a unicornuate uterus; meanwhile, a unicornuate uterus with a rudimentary horn can result from failure of canalization of the ducts, while a bicornuate or didelphys uterus can result from the failure of the Müllerian ducts to fuse [14].

The rudimentary horn has been linked with a high incidence of endometriosis, cornual pregnancy, and dysmenorrhea [14]. Urinary tract anomalies are often associated more with a unicornuate uterus compared to other Müllerian duct anomalies, with contralateral renal agenesis being the most commonly reported abnormality [8]. Renal anomalies are present in up to 40.5% of patients with a unicornuate uterus [6]. This is due to the identified embryological connection between the Müllerian and Wolffian systems [15]. Whether right-sided unicornuate uteri occur more often than left-sided ones remains unclear; while some studies report the incidence rate of right-sided rudimentary uterine horns as 80%, other reports indicate the incidence rate is 62% [11]. However, in our case, the patient presented with the left-sided type. In our case, both MRI and 3D ultrasonography were performed and provided a detailed anatomic structure of the uterine surface, endometrial cavity, adnexa, and other organs such as the kidneys. Hysterosalpingograms can be useful but were not applicable to our patient, who was still a virgin. Proper gynecological examinations are essential to accurately identify the condition and provide appropriate management. Regarding imaging modalities, two-dimensional ultrasound (US2D) can be used initially because it is non-invasive, simple, and low-cost; however, its accuracy is highly dependent on the examiner's experience [16]. Alternatively, three-dimensional ultrasound (US3D) has higher interobserver reliability, provides more reliable images, and allows for the assessment of the vagina and cervix; however, It is less accessible and demands more specialized training in comparison to US2D [17]. The gold standard for diagnosing Müllerian duct anomalies is considered to be MRI. It provides reliable and objective tridimensional information about all peritoneal and genital anatomy, with the exception of the tubes, and it is applicable in all clinical contexts, including those characterized by obstructive malformations. However, it is less accessible than ultrasound, requires more level of expertise to interpret the results, and is more expensive [5,18]. Hysterosalpingo-contrast-sonography is a minimally invasive technique that provides useful information regarding the uterine cavity and the cervix; however, its accuracy is operator-dependent. Additionally, the distention of the uterine cavity may alter the internal contours, potentially resulting in false-negative findings [19]. Based on anatomic variations, the European Society of Human Reproductive (ESHR)/European Society of Gynecological Endoscopy have classified congenital anomalies of the female genital tract as follows: class U0, normal uterus; class U1, dysmorphic uterus; class U2, septate uterus; class U3, all fusion defects including a bicorporeal uterus; class U4, hemi-uterus or unilateral uterus; class U5, aplastic uterus; class U6, unclassified uterine malformation. Class U4 is subdivided into types a and b. Class U4a represents a uterus with a communicating or non-communicating rudimentary cavity; class U4b indicates a uterus without a rudimentary cavity [16,20]. In our case, the patient presented with an ESHR class U4a anomaly.

A unicornuate uterus requires surgical intervention only if there is a cavitated, non-communicating rudimentary uterine horn, which must be resected because of the pain caused by the obstruction of menstrual flow, and in some cases, a reduction in its muscular mass can lead to isthmus-cervical incompetence, necessitating cerclage in a future pregnancy [21]. The advancement of minimally invasive surgery enables laparoscopic or hysteroscopic treatment in most cases [22,23]. Laparoscopic resection of the non-communicating rudimentary horn can relieve pelvic pain and dysmenorrhea, optimize fertility, and prevent endometriosis, miscarriage, and ectopic pregnancy. Thus, due to these reasons, it is the recommended treatment of choice [11]. Other methods such as surgical connection to the non-communicating uterine horn using hysteroscopy or endometrial ablation have also been reported to be successful [24,25]. 

## Conclusions

A unicornuate uterus with a non-communicating rudimentary horn is a rare condition. However, it should be considered in suspicious cases such as those involving acute abdominal pain and progressive dysmenorrhea, particularly in patients who have a history of recurrent visits to healthcare centers for injectable pain relievers. The early diagnosis of such a condition is crucial to prevent any complications. A thorough preoperative assessment for identifying the type of Müllerian duct anomaly and potential urinary tract anomaly is an extremely significant step in preventing intraoperative complications. Advanced laparoscopic skill levels are important for the effective management of this condition. Future research should focus on optimizing early diagnostic strategies and refining laparoscopic management techniques.
